# A digitalized nosocomial risk management framework during the COVID-19 pandemic: practice-based insights from a tertiary cancer hospital in Southwest China

**DOI:** 10.3389/fpubh.2026.1925119

**Published:** 2026-10-12

**Authors:** Qingqing Tian, Qin Huang, Jiayang Tang, Anran Liu, Hui Wang, Weiwei Yang, Chunlin Wu

**Affiliations:** Department of Hospital Infection Control and Public Health, Sichuan Cancer Hospital, Chengdu, China

**Keywords:** cancer hospital, COVID-19, digital surveillance, nosocomial infection control, post-pandemic preparedness, Questionnaire Star, R programming, risk management

## Abstract

**Background:**

Cancer patients represent a particularly vulnerable population during infectious disease outbreaks, with substantially elevated risk of infection and mortality compared to the general population. Comprehensive nosocomial risk management frameworks for specialized oncology hospitals remain under-documented in the literature.

**Methods:**

This community case study describes a comprehensive, multi-component epidemic risk management program implemented at Sichuan Cancer Hospital—the largest tertiary cancer hospital in Southwest China—from January 2020 to September 2022. The program integrated seven management pillars with a digital risk-reporting pipeline built on Questionnaire Star^®^ and R programming language.

**Results:**

Over the 32-month study period, the hospital achieved a 98% staff vaccination rate with 95% booster coverage. Following its deployment in August 2022, during the final weeks of the study period, the digital reporting system processed risk-investigation data and generated actionable summary tables within 5 min of submission. No nosocomial COVID-19 cases were detected among patients or staff during the surveillance period.

**Conclusion:**

The experience demonstrates that systematic integration of management innovations with digital technologies can support hospital infection prevention even under extreme epidemiological pressure; the absence of detected nosocomial cases, however, cannot be causally attributed to the program alone given concurrent community-level restrictions. The framework’s pathogen-agnostic architecture supports transferability to future respiratory virus epidemics in cancer hospital settings.

## 1 Introduction

The coronavirus disease 2019 (COVID-19) pandemic, caused by severe acute respiratory syndrome coronavirus 2 (SARS-CoV-2), was first identified in Wuhan, China, in December 2019 and rapidly evolved into a global public health crisis, with the World Health Organization declaring a pandemic on March 11, 2020 (1, 2). The high transmissibility of SARS-CoV-2, with reproduction numbers varying across countries and prevention scenarios (3), and the clinical severity documented among early hospitalized cohorts (4), underscored the urgency of robust infection prevention. As of early 2023, COVID-19 had caused over 760 million confirmed cases and 6.9 million deaths worldwide.

Cancer patients constitute a particularly high-risk group in the context of COVID-19. Immunosuppression resulting from both the malignancy itself and anticancer treatments (chemotherapy, radiotherapy, and immunotherapy) elevates susceptibility to infection, while frequent hospital visits for treatment create repeated exposure opportunities. Studies have consistently reported that cancer patients with COVID-19 experience higher rates of severe illness, intensive care unit admission, and mortality compared to the general population (5–9).

Despite the extensive literature on COVID-19 clinical outcomes in cancer patients, structured descriptions of comprehensive risk management frameworks implemented in oncology hospitals remain scarce. Most published reports focus on isolated interventions—such as triage protocols (10, 11), vaccination campaigns (12, 13), or telehealth adoption (14)—rather than integrated, multi-component programs. Furthermore, the role of digital tools in automating risk-investigation workflows within cancer hospitals has received limited attention (15–17).

This community case study presents the design, implementation, and outcomes of a comprehensive epidemic risk management program at Sichuan Cancer Hospital, the largest specialized oncology center in Southwest China. The program was specifically developed to address a critical gap in the literature: the absence of an integrated, replicable framework that combines organizational management strategies with a digital data-processing pipeline for nosocomial risk management in a specialized cancer hospital setting under pandemic conditions.

## 2 Context

### 2.1 Institutional setting

Sichuan Cancer Hospital, affiliated with the School of Medicine of the University of Electronic Science and Technology of China, is the largest specialized cancer hospital in Southwest China and was the first tertiary-A cancer hospital established in the region. The hospital has approximately 1,600 open beds, over 4,000 healthcare workers, and serves a catchment population of over 80 million across Sichuan Province. As a regional referral center, it receives patients from all 21 prefecture-level divisions of Sichuan and neighboring provinces, creating a complex nosocomial infection risk landscape—particularly during infectious disease outbreaks where patients travel across administrative boundaries for continued cancer treatment.

### 2.2 Epidemiological context in Sichuan Province

The COVID-19 outbreak in Sichuan Province was characterized in a detailed epidemiological analysis that documented the transmission dynamics and the impact of public health interventions during the initial wave (18). Chengdu, the provincial capital with a population exceeding 21 million, experienced multiple local outbreaks throughout 2020–2022, with the most severe occurring in August–September 2022, driven by the highly transmissible Omicron BA.5 sub-lineage (19). This outbreak resulted in city-wide lockdowns and placed enormous pressure on the hospital’s ability to maintain essential cancer services while simultaneously preventing nosocomial transmission.

### 2.3 Identified needs and rationale for innovation

Prior to the pandemic, the hospital’s infection surveillance relied primarily on manual reporting channels (telephone, WeChat messages, paper forms), which proved inadequate under pandemic conditions characterized by rapidly changing risk categories, large volumes of daily reports, and the need for near-real-time situational awareness. Three specific needs were identified: (1) an organizational leadership structure capable of rapid, coordinated decision-making; (2) a risk-stratified management framework that could be dynamically adjusted as epidemiological conditions evolved; and (3) a digital toolchain to automate the collection, processing, and dissemination of risk-investigation data. These needs drove the design of the comprehensive program described in this report.

## 3 Program description: key elements of the risk management framework

### 3.1 Management enhancement strategies

#### 3.1.1 Leadership structure and accountability mechanism

A Hospital Leadership Group for Coping with COVID-19 was established at the onset of the pandemic, co-chaired by the Party Secretary and the Dean. This dual-leadership model ensured shared accountability between administrative and clinical leadership and enabled rapid cross-departmental coordination. The group convened daily briefings during outbreak periods to review epidemiological intelligence, adjust prevention protocols, and allocate resources. This structure also established a direct reporting line from departmental infection control teams to the hospital executive level, bypassing intermediate administrative layers that could introduce decision-making delays.

#### 3.1.2 Zoned and tiered prevention and control

A four-tier zoning system (general, Level 1, Level 2, and Level 3) was implemented across all hospital departments, with each tier corresponding to specific prevention and control protocols calibrated to the department’s assessed risk of exposure. Risk levels were dynamically adjusted based on real-time epidemiological intelligence, allowing the hospital to escalate or de-escalate protocols without disrupting essential cancer care services. Physical barriers, dedicated patient pathways, and zoning-specific personal protective equipment (PPE) requirements were defined for each tier.

#### 3.1.3 Dynamic epidemiological intelligence

The hospital invested in building a professional epidemiological team by recruiting public health specialists dedicated to real-time epidemic risk monitoring. This team maintained continuous surveillance of domestic and international COVID-19 developments, monitored local transmission patterns, and provided daily risk assessments to the leadership group. The intelligence outputs directly informed zoning adjustments, screening protocol modifications, and resource allocation decisions.

#### 3.1.4 Three-level network supervision and grid-based inspection

A three-tier network supervision structure—comprising the Hospital Infection Committee, the Hospital Infection Management Office, and departmental Infection Control Teams—was established to enforce compliance with prevention protocols. Daily grid-based inspections were conducted across all clinical and non-clinical departments, with findings reported through a standardized digital checklist and escalated to the leadership group when deficiencies were identified.

#### 3.1.5 Network-based education and training mechanism

Recognizing that staff knowledge and skills form the foundation of effective risk identification, the hospital developed a comprehensive network-based education and training mechanism for COVID-19 prevention and control (Figure 1). The mechanism comprised online learning modules, video-conference training sessions, and a continuously updated infection control manual. Training content was stratified by staff role (clinical, nursing, administrative, support) and competency requirements. A pre- and post-training assessment system tracked knowledge acquisition, with remedial training mandated for staff who did not meet the competency threshold.

**Figure 1:**
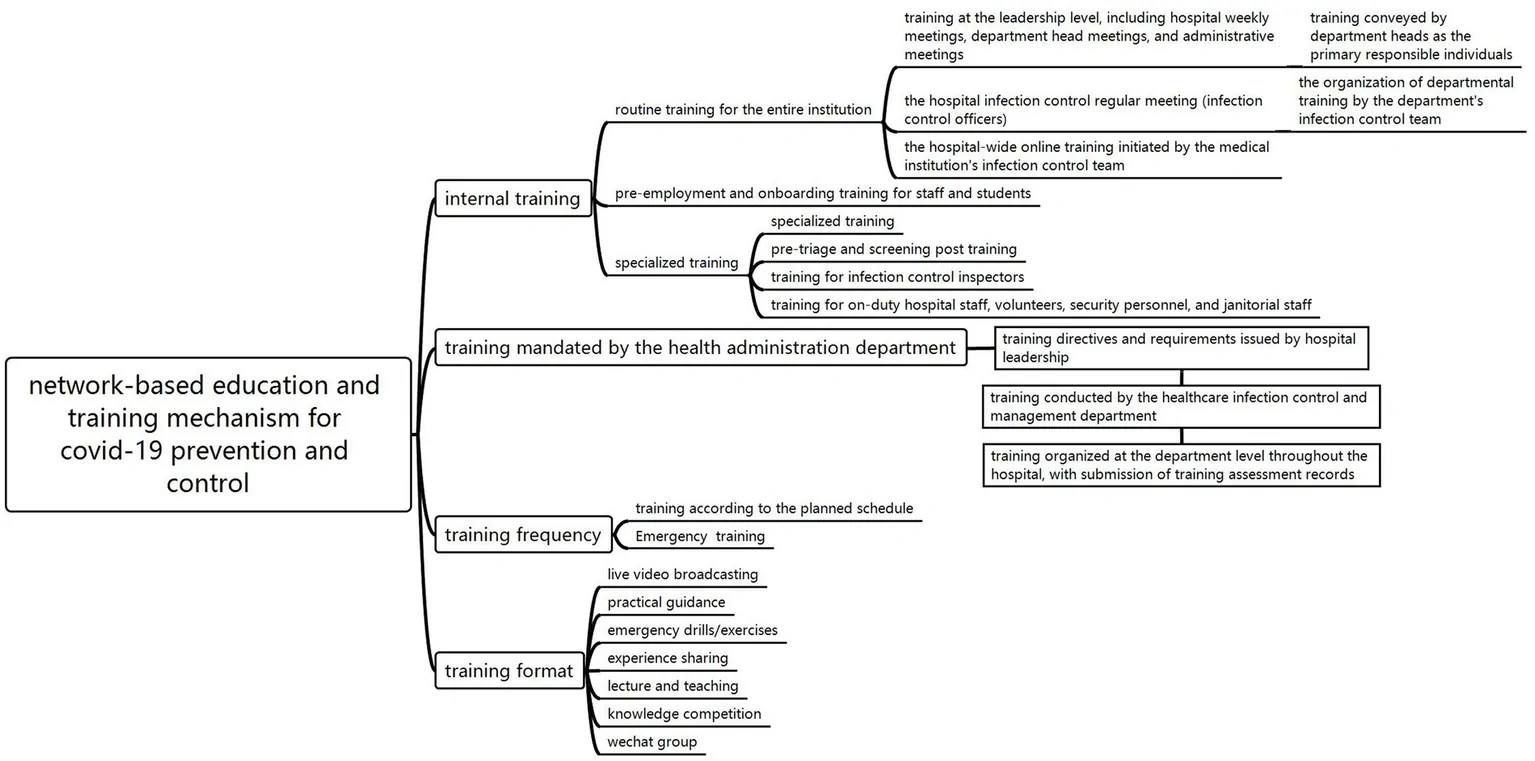
Network-based education and training mechanism for COVID-19 prevention and control.

#### 3.1.6 Vaccination campaign to establish an immunity barrier

The hospital adopted an assertive vaccination promotion strategy aligned with the national “should vaccinate as much as possible” policy for healthcare workers. Vaccination and booster doses were administered on-site through the hospital’s occupational health service, removing access barriers. By September 2022, 98% of hospital staff had completed the primary vaccination series and 95% had received a booster dose.

The seven management pillars described, together with their primary objectives, key actions, responsible personnel, and reported outcomes (classified in Table 1 as measured indicators or operational objectives), are summarized in Table 1. These components were introduced progressively rather than simultaneously. The leadership structure, zoned-tiered prevention, epidemiological intelligence, and grid-based supervision were established during the first months of 2020 in response to the initial outbreak; the network-based education and training mechanism was launched in January 2020; the vaccination campaign scaled progressively from early 2021 as vaccines became available and reached full coverage by September 2022; and the digital risk-reporting pipeline was developed and deployed in August 2022 in direct response to the Chengdu Omicron BA.5 outbreak (Figure 2). Figure 2 presents a timeline showing when each component was introduced, modified, and fully implemented in relation to local epidemic activity. It should be noted that the digital component therefore operated only during the final weeks of the 32-month surveillance period; the absence of detected nosocomial cases over the full period reflects the combined operation of the organizational components throughout the study window and of the digital pipeline during the period of highest transmission pressure.

**Table 1:** Seven management pillars of the nosocomial epidemic risk management program.

Management pillar	Primary objective	Key actions	Responsible personnel	Measured indicator/operational objective
1. Leadership accountability	Establish unified command structure	Co-chaired by Party Secretary and Dean; daily briefings	Hospital Leadership Group	Operational objective: zero-delay decision-making chain
2. Zoned-tiered prevention	Risk-stratified departmental protocols	Four-tier system (general, L1, L2, L3); dynamic adjustments	Department Heads and Infection Control Teams	Operational objective: no cross-contamination between zones
3. Epidemiological intelligence	Real-time risk monitoring	Dedicated public health team; continuous surveillance of domestic/international outbreaks	Epidemiology Team	Operational objective: early warning 24–48 h ahead of community outbreaks
4. Grid-based supervision	Three-level network enforcement	Committee → Office → Department teams; daily inspections	Infection Control Committee and Office	Measured indicator: 100% daily inspection coverage
5. Network-based training	Ensure staff competency	Online modules; video conferences; infection control manual	Training and Education Working Group	Measured indicator: 98% training completion rate
6. Vaccination campaign	Establish immunity barrier	Promoted per national policy; on-site administration	Occupational Health and Infection Control	Measured indicator: 98% vaccination rate; 95% booster coverage
7. Digital risk-reporting pipeline	Automate risk-investigation workflow	Questionnaire Star + R programming; <5 min processing	Infection Control Office and IT Department	Measured indicator: >5,000 risk records processed with zero backlog

The final column distinguishes quantitatively measured indicators from qualitative operational objectives, as defined in Section 3.1.6.

**Figure 2:**
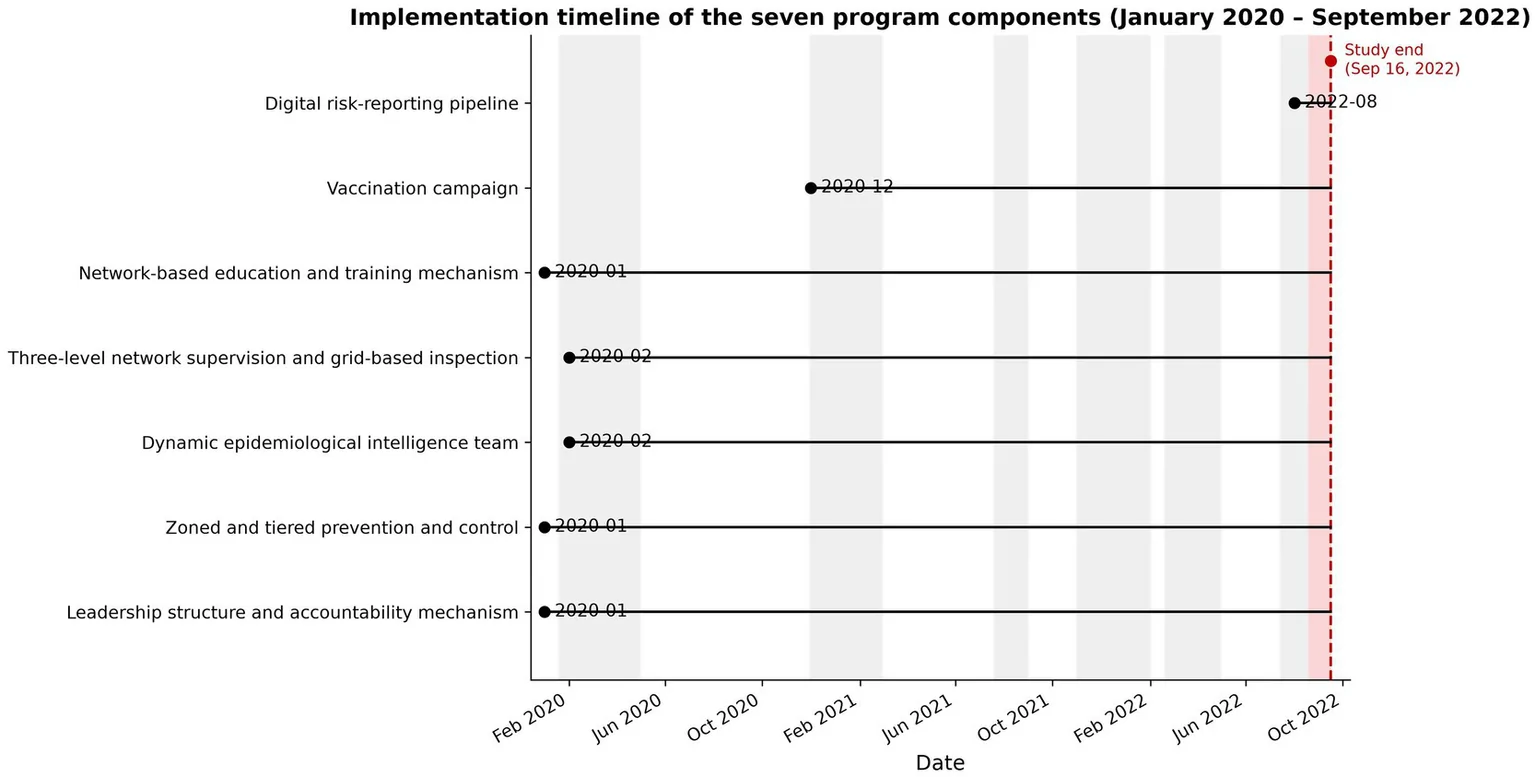
Implementation timeline of the nosocomial epidemic risk management program (January 2020–September 2022). Bars indicate the period during which each component was introduced, modified, or fully implemented in relation to local epidemic activity (grey shading) and the August 2022 Chengdu Omicron BA.5 outbreak (red band). The digital risk-reporting pipeline was deployed in August 2022 and therefore operated only during the final weeks of the study window.

Operational indicator definitions. The outcomes reported in Table 1 comprise both measured indicators and operational objectives. Measured indicators include: the vaccination rates (98% primary series, 95% booster), derived from the hospital occupational health vaccination registry as of September 2022; the training completion rate (98%), derived from the online learning platform’s assessment records; the daily inspection coverage (100%), recorded through the standardized digital inspection checklist during the observation period; and the processing time (<5 min) and number of records processed (>5,000 with zero backlog), derived from the automated processing log of the digital pipeline during August–September 2022. Indicators such as ‘zero-delay decision-making chain’, ‘no cross-contamination between zones’, and ‘early warning 24–48 h ahead of community outbreaks’ represent documented operational objectives and qualitative process records rather than quantitatively measured outcomes; they are reported to describe intended system performance and should be interpreted accordingly.

### 3.2 Technological innovation: the digital risk-reporting pipeline

The intensification of the pandemic in 2022—driven by highly transmissible Omicron sub-lineages (19)—exposed critical limitations of traditional risk-reporting channels. During the August 2022 Chengdu outbreak, manual telephone- and WeChat-based reporting channels were overwhelmed by the volume of daily risk-investigation requests, creating reporting backlogs that delayed risk stratification. In response, in August 2022 the hospital developed a four-step digital risk-reporting pipeline: (1) end-user submits risk data via mobile device to the Questionnaire Star^®^ server; (2) authorized infection control officers export raw data as an Excel (.xlsx) file from the platform’s administrative backend; (3) an R script reads and imports the Excel file, performs automated data cleaning (date validation, deduplication), strips personal identifiers, and generates three summary tables; (4) summary tables are output within 5 min of the original submission.

#### 3.2.1 Component 1: structured data capture via Questionnaire Star^®^

Questionnaire Star^®^ (Changsha Ranxing Information Technology Co., Ltd., China)—a widely used commercial online survey platform compliant with China’s data security regulations—was configured as the front-end data capture interface. The risk-reporting form comprised 17 structured mandatory fields, including demographic information, personnel category, health code status, nucleic acid test results, residential risk classification, and close-contact history. The form was distributed via QR code and WeChat links, enabling mobile access for hospital staff and affiliated personnel (Figure 3a).

**Figure 3:**
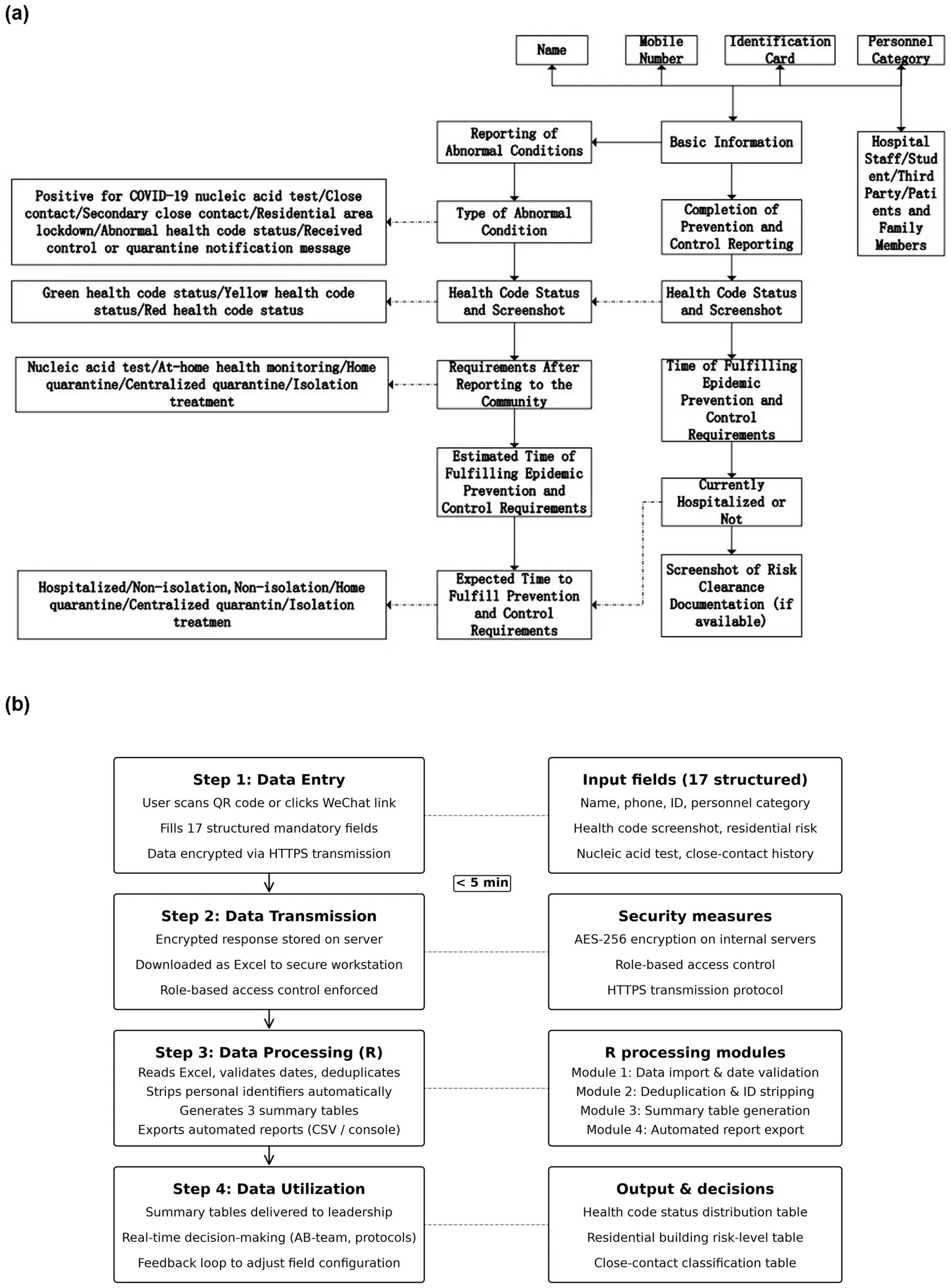
The digital risk-reporting workflow. **(a)** Structured risk-reporting fields configured in Questionnaire Star^®^; **(b)** the four-step digital risk-reporting data pipeline.

While Questionnaire Star^®^ was selected for its regulatory compliance and local technical support within the Chinese healthcare data environment, functionally equivalent platforms are available internationally, including REDCap (Research Electronic Data Capture), Qualtrics, Google Forms, Microsoft Forms, and LimeSurvey. The R data-processing logic is platform-agnostic: it accepts any structured CSV or Excel file as input, regardless of the front-end survey tool used. Institutions in other regulatory environments may substitute the capture platform while preserving the back-end automated pipeline architecture, a key consideration for international transferability (17, 20).

All data transmission between end-user devices and the Questionnaire Star^®^ server occurs over HTTPS (TLS 1.2+) encryption. On the server side, stored responses are protected by AES-256 encryption at rest. After export, data reside on a dedicated, password-protected workstation within the hospital’s internal network, accessible only to designated infection control personnel through role-based access control. The R script strips all direct personal identifiers—name, phone number, national identification number, IP address, and uploaded screenshots (health code, nucleic acid test reports, risk-clearance certificates)—before any summary tables are generated or shared. This de-identification pipeline aligns with China’s Personal Information Protection Law (PIPL) and is conceptually consistent with relevant data-protection principles, including elements of the EU General Data Protection Regulation (GDPR) and the US Health Insurance Portability and Accountability Act (HIPAA) (21). It should be noted that no formal legal or technical compliance assessment against GDPR or HIPAA was performed.

#### 3.2.2 Component 2: automated data processing via R programming language

R programming language (version 4.1.3; R Foundation for Statistical Computing, Vienna, Austria) was employed as the back-end data processing engine. A custom R script was developed to perform the following automated operations: (a) data import from Excel with standardized variable naming and labeling; (b) immediate removal of all direct personal identifiers (name, phone number, national identification number, IP address, and uploaded screenshot fields) from the analytical dataset, retaining only an anonymized deduplication key; (c) date validation, with missing or invalid completion dates treated as missing rather than imputed; (d) filtering for abnormality reports followed by deduplication that retains the most recent record per individual; and (e) generation of three summary tables: health-code status distribution, residential building risk-level distribution, and close-contact classification. The complete R script (Supplementary material 1) reproduces this workflow exactly as described. The complete pipeline is illustrated in Figure 3b. The complete R script is provided as Supplementary material 1 (16).

#### 3.2.3 Operational performance during the August 2022 Chengdu outbreak

The digital pipeline underwent its most rigorous stress test during the local COVID-19 outbreak in Chengdu commencing on August 25, 2022. During this period, the system processed a surge in risk-investigation submissions from hospital staff, with end-to-end processing time—from user submission to summary table delivery—consistently under 5 min. Processing time was measured for each daily batch as the interval between submission of the final record in that batch and completion of the summary tables by the R script, as recorded in the automated processing log; the reported value represents the typical (median) duration across daily runs during this period rather than a single best-case measurement. This performance represented a substantial improvement over the previous manual workflow, which required hours of transcription, calculation, and cross-referencing; direct quantitative comparison with the manual procedure was not formally recorded and is therefore reported as a qualitative improvement. The rapid availability of risk-stratified data enabled the leadership group to make timely decisions regarding staff deployment, including the implementation of an alternate-team (AB-team) staffing model.

## 4 Program outcomes

### 4.1 Epidemic surveillance data (January 2020–September 2022)

Over the 32-month surveillance period (January 23, 2020–September 16, 2022), the hospital’s pre-examination triage system processed a progressively increasing volume of patients as medical services were restored following the initial lockdown. The triage system screened all entering individuals for fever and epidemiological history, generating time-series data across three population groups: outpatients and visitors, hospitalized patients, and healthcare workers (Figure 4). Among outpatients and visitors, triage visit volumes exhibited a seasonal pattern with peaks in summer and autumn, reflecting both the resumption of cancer treatment activities and the local epidemic dynamics. Fever-clinic consultations followed a similar seasonal pattern, with 1,722 visits recorded over the surveillance period and peak activity in summer and autumn.

**Figure 4:**
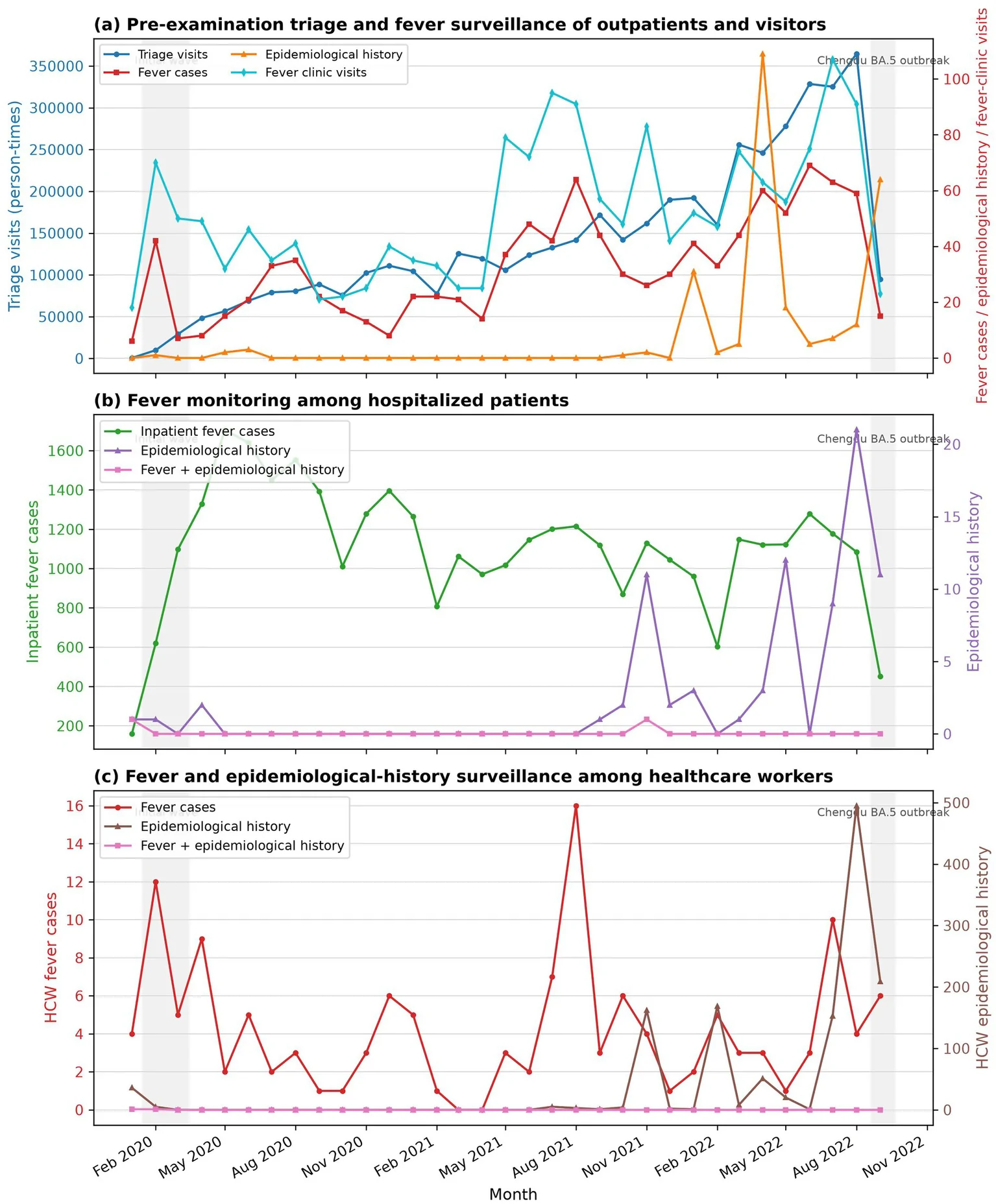
Monthly aggregated epidemic surveillance time series (January 2020–September 2022). **(a)** Pre-examination triage, fever-clinic, and fever/epidemiological-history surveillance of outpatients and visitors; **(b)** fever and epidemiological-history monitoring among hospitalized patients; **(c)** fever and epidemiological-history surveillance among healthcare workers. Shaded bands indicate major community outbreak periods in Chengdu. In each panel, the left-hand axis shows the primary volume indicator [triage visits in panel **(a)**; fever counts in panels **(b,c)**] and the right-hand axis shows the remaining indicators, as labeled in each panel.

Among the hospital workforce (over 4,000 personnel), fever reporting followed the same major-peak-in-summer-autumn, minor-peak-in-winter–spring seasonal pattern, with the highest monthly fever count recorded during the August 2022 Omicron BA.5 outbreak. Epidemiological history reporting among healthcare workers showed distinct peaks corresponding to community transmission events, demonstrating the sensitivity of the surveillance system to external epidemiological dynamics.

### 4.2 Achievement of zero detected nosocomial infection

The most critical program outcome was the absence of detected nosocomial COVID-19 cases within the hospital throughout the entire 32-month surveillance period. By September 16, 2022, cumulative statistics showed zero detected cases of hospital-acquired COVID-19 among both inpatients and healthcare workers. This outcome was observed despite the hospital’s location in a metropolitan area that experienced multiple community outbreaks, including the severe Omicron BA.5 wave in August–September 2022. Given the testing strategy described below, we use the term “no detected nosocomial COVID-19 cases” rather than “zero nosocomial transmission” to describe this outcome.

Case definition and ascertainment. A nosocomial COVID-19 case was defined as a SARS-CoV-2 infection, confirmed by real-time reverse transcription polymerase chain reaction (RT-PCR), in (i) an inpatient who developed symptoms or had a first positive test 48 h or more after admission, or (ii) a healthcare worker or affiliated personnel whose infection was epidemiologically linked to in-hospital exposure rather than to household or community transmission. Community-acquired and hospital-acquired infections were distinguished through structured epidemiological interviews assessing exposure history, symptom onset relative to admission, and contact with confirmed cases in the community or household.

Surveillance methods. Testing was triggered by (i) symptoms consistent with COVID-19 identified at pre-examination triage or during hospitalization; (ii) identification as a close or secondary contact of a confirmed case; and (iii) entry into hospital zoned risk areas. Consistent with national protocols, close contacts of any confirmed case (in the community or within the hospital) were identified within 24 h, quarantined, and tested. Of note, routine systematic screening for asymptomatic infection was not performed throughout the entire study period; therefore, the surveillance strategy may not have detected all asymptomatic infections.

Denominators. The surveillance system generated the following denominators over the 32-month observation period: 4,593,269 pre-examination triage encounters (including all patients, visitors, and staff entering the hospital); 36,430 inpatient fever episodes; 1,722 fever-clinic visits; and 138 fever reports and 1,325 epidemiological-history reports among the hospital workforce (over 4,000 healthcare workers under surveillance). The digital pipeline processed more than 5,000 risk-investigation records during the August 2022 outbreak.

## 5 Discussion

This community case study describes a comprehensive nosocomial epidemic risk management program implemented at a large tertiary cancer hospital during the COVID-19 pandemic, yielding three principal findings: (1) the systematic integration of management innovations with digital technologies can function as a force multiplier for infection prevention; (2) the framework’s core architecture is pathogen-agnostic and transferable to future epidemic scenarios; and (3) no nosocomial COVID-19 cases were detected over 32 months despite repeated community outbreaks, a finding best interpreted as an association between the program and the absence of detected cases rather than proof of causation.

### 5.1 Integration of management and technology as a force multiplier

The program’s key innovation lies not in any single component but in the systematic integration of management strategies with digital technologies. The seven management pillars—leadership accountability, zoned-tiered prevention, epidemiological intelligence, grid-based supervision, network-based training, vaccination campaigns, and the digital pipeline (Table 1)—are individually well-documented in the infection prevention literature (11, 22–24), but their combined, mutually reinforcing implementation has not been described in a cancer hospital context. The digital pipeline in particular served as the informational backbone of the framework, enabling the rapid translation of raw risk-investigation data into actionable intelligence that directly informed management decisions. This finding aligns with recent systematic reviews showing that hospitals employing integrated, multi-component infection prevention bundles achieve superior outcomes compared to those implementing isolated interventions (24, 25). Layered multimodal quality-improvement initiatives have likewise demonstrated measurable reductions in device-associated infections in intensive care settings (26), further supporting the value of integrated rather than isolated interventions.

### 5.2 Transferability beyond COVID-19

Although the program was developed and tested in the COVID-19 context, its core architecture is pathogen-agnostic. The structured data-capture templates in Questionnaire Star^®^ can be rapidly reconfigured for other infectious disease threats (e.g., influenza, respiratory syncytial virus, or novel respiratory pathogens) by modifying the field set without altering the R processing logic. The R script’s modular design allows the addition of new data validation rules and the generation of pathogen-specific summary tables with minimal reprogramming. International equivalents of the data-capture platform [REDCap, Qualtrics, Google Forms (20)] and the platform-agnostic nature of the R back end (which accepts any structured CSV or Excel input) facilitate cross-border replication. This flexibility is particularly relevant given the increasing recognition that future respiratory virus epidemics are inevitable and that healthcare systems must maintain “warm” surveillance infrastructure between outbreaks (27, 28).

### 5.3 Comparison with published literature

The existing literature on COVID-19 infection prevention and control in oncology settings can be broadly categorized into three groups: (a) clinical outcome studies reporting infection rates and mortality among cancer patients (5–9, 29); (b) expert commentaries and guideline documents providing management recommendations (10, 30–33); and (c) technology-focused reports on specific tools such as telehealth or health informatics systems (11, 14, 15). The present study contributes to a fourth, under-represented category: structured, practice-based descriptions of integrated frameworks that combine organizational and technological components into a coherent whole. Recent publications have emphasized the importance of such integrated approaches for post-pandemic preparedness (25–27), but detailed implementation accounts from specialized cancer hospitals remain limited. The R-based automated processing pipeline described here extends the existing literature on digital surveillance tools (15, 16, 25) by demonstrating a low-cost, platform-agnostic solution that does not require proprietary hospital information system integration.

### 5.4 Practical challenges and recommendations

Several challenges encountered during program implementation warrant discussion. First, information interoperability between medical institutions remains a systemic barrier: different hospitals in the same region used incompatible reporting systems, preventing cross-institutional risk data sharing. This limitation could be addressed through the adoption of standardized data exchange formats (e.g., HL7 FHIR) at the policy level. Second, the reliance on Questionnaire Star^®^ as a commercial platform introduces a vendor-dependency risk; the platform-agnostic design described in Section 3.2.1 provides a mitigation strategy. Third, the initial configuration of the 17-field form required multiple iterations to balance comprehensiveness with completion time—a consideration for institutions developing similar tools. Finally, staff compliance with daily risk-reporting was initially suboptimal and required targeted communication campaigns and integration of reporting status into departmental performance metrics.

### 5.5 Limitations

This report has several limitations that should be acknowledged. First, as a single-center case study, the generalizability of the findings to other institutional contexts—particularly hospitals with different resource levels, regulatory environments, or patient populations—requires further validation through multi-center replication studies. Second, the program was implemented during China’s “Zero-COVID” policy period, which included community-level non-pharmaceutical interventions (NPIs) such as lockdowns, mass testing, and travel restrictions. The observed absence of detected nosocomial cases reflects the combined effect of the hospital’s internal program and external community-level NPIs; isolating the independent contribution of the hospital’s program is not possible from this observational data. Future studies conducted in settings without concurrent community-level restrictions would help estimate the program’s standalone effectiveness. Third, asymptomatic infections may have gone undetected, as routine asymptomatic screening of all staff and patients was not systematically performed during the entire study period. Fourth, the 32-month observation window, while capturing multiple epidemic waves, does not include the full post-pandemic transition period. Finally, this report is observational in nature and was not designed as a controlled intervention study with a comparator group.

### 5.6 Interaction with community-level Zero-COVID policy

An important interpretative consideration is the interaction between the hospital’s internal program and China’s national Zero-COVID policy, which was active during the entire study period. Community-level non-pharmaceutical interventions (NPIs)—including city-wide lockdowns during the August 2022 Chengdu outbreak, universal masking mandates, mass PCR testing, and inter-city travel restrictions—undoubtedly reduced the baseline risk of SARS-CoV-2 introduction into the hospital. However, several observations are consistent with a contribution of the hospital’s internal program to the absence of detected nosocomial cases. First, the hospital continued to receive patients from across Sichuan and neighboring provinces throughout the study period, including from regions with active community transmission, because cancer treatment cannot be indefinitely deferred. Second, the August 2022 Chengdu outbreak occurred despite existing community-level NPIs, indicating that community measures alone could not completely eliminate importation risk. Third, the digital pipeline was activated specifically in response to the failure of manual reporting channels during this outbreak, and the rapid availability of risk-stratified data directly enabled the leadership group to implement targeted, rather than blanket, staff deployment decisions—suggesting that the program may have added value beyond what community measures alone could provide. A cautious interpretation is therefore that the absence of detected nosocomial cases represents the combined effect of community-level NPIs and the hospital’s internal framework, with each component addressing different points in the transmission chain.

### 5.7 Post-study implementation example: influenza preparedness (2023–2024)

Following the lifting of Zero-COVID restrictions in December 2022, and outside the stated study window (January 2020–September 2022), the hospital repurposed the digital risk-reporting pipeline for the 2023–2024 influenza season. The Questionnaire Star^®^ form was reconfigured within 48 h by replacing COVID-19-specific fields (health code status, nucleic acid test results) with influenza-specific fields (influenza vaccination status, antiviral prophylaxis, symptom checklist), while preserving the demographic and contact-tracing fields. The R script required modification to only the variable-labeling module (approximately 20 lines of code), with the core data-cleaning, deduplication, and summary-table-generation logic remaining unchanged. This post-study implementation example demonstrates the framework’s operational flexibility and illustrates the feasibility of adapting the pathogen-agnostic architecture described in Section 5.2 to a different respiratory pathogen. Because this period falls outside the stated study window and systematic outcome data were not collected, it is reported as an implementation example rather than as a formal validation of the framework.

## 6 Conclusion

This community case study demonstrates that a systematic, multi-component epidemic risk management program—integrating organizational strategies with a digital surveillance pipeline—was associated with an absence of detected nosocomial COVID-19 cases in a large tertiary cancer hospital, even under extreme epidemiological pressure. The framework’s pathogen-agnostic architecture, platform-agnostic data-processing logic, and modular R-based automation pipeline provide a replicable template for cancer hospitals and other specialized healthcare facilities preparing for future respiratory virus epidemics. While the absence of detected nosocomial cases must be interpreted in the context of concurrent community-level interventions during China’s Zero-COVID period, the program’s operational performance—particularly the <5-min data-processing capability and the successful repurposing for influenza surveillance—supports its practical utility. Multi-center replication studies in diverse regulatory and resource settings are needed to evaluate generalizability and to isolate the program’s independent contribution to nosocomial infection prevention.

## 7 Acknowledgment of conceptual and methodological constraints

This study is subject to the following conceptual and methodological constraints: (1) the single-center design limits generalizability; findings should be validated in multi-center studies across diverse institutional and regulatory contexts. (2) The observational nature precludes causal attribution of the absence of detected nosocomial cases to specific program components. (3) The Zero-COVID policy period during which the program operated confounds the estimation of standalone program effectiveness. (4) Asymptomatic SARS-CoV-2 infections may have been undetected due to the absence of systematic asymptomatic screening. (5) The 32-month observation window does not capture the complete post-pandemic transition, and longer-term sustainability assessments are needed. (6) The digital pipeline’s performance was evaluated during a single hospital’s operations; scalability to multi-hospital networks remains untested. (7) Because systematic asymptomatic screening was not performed throughout the study period, the reported outcome refers to detected cases only.

## Data Availability

The datasets presented in this study can be found in online repositories. The names of the repository/repositories and accession number(s) can be found in the article/Supplementary material.

## Supplementary material

SUPPLEMENTARY MATERIAL 1 The complete R programming script for the automated risk-population data-processing pipeline described in Section 3.2.2.

SUPPLEMENTARY MATERIAL 2 The de-identified aggregated surveillance dataset in Excel (.xlsx) format underlying the epidemic curves presented in Section 4.1.
