# Supplementary material for: A digitalized nosocomial risk management framework during the COVID-19 pandemic: practice-based insights from a tertiary cancer hospital in Southwest China
Source: Front Public Health. 2026 Oct 12;14:1925119. doi: 10.3389/fpubh.2026.1925119 (PMC13696459; doi:10.3389/fpubh.2026.1925119)
Supplement: Supplementary file 1 [file Supplementary_file_2.docx]

**Supplementary Material**

**R Programming Code for the Automated Risk-Population Data-Processing Pipeline**

Associated Manuscript: "A Digitalized Nosocomial Risk Management Framework During the COVID-19 Pandemic: Practice-Based Insights from a Tertiary Cancer Hospital in Southwest China"

Journal: Frontiers in Public Health

**1. Overview**

This supplementary document contains the complete R programming script (version 4.1.3) used to process risk-population data exported from the Questionnaire Star® online survey platform at Sichuan Cancer Hospital. The pipeline performs automated data cleaning, de-identification, deduplication, and summary statistics generation for hospital-wide COVID-19 risk surveillance.

The script is structured into four sequential modules: (i) package installation and library loading, (ii) data import and variable labeling, (iii) data cleaning and de-identification (removal of direct personal identifiers immediately after import, date validation, filtering, and deduplication), and (iv) automated summary table generation. The complete pipeline executes within seconds and was used daily to support real-time decision-making during the COVID-19 pandemic.

**2. Dependencies**

The script requires R version 4.0 or higher with the following packages installed:

• Hmisc (version 4.7-0 or higher) — variable labeling and data description

• lubridate (version 1.9-0 or higher) — date parsing and manipulation

• tidyverse (version 1.3-2 or higher) — data manipulation (dplyr, tidyr)

• readxl (version 1.4-0 or higher) — reading Excel (.xlsx) data files

**3. Input Data Format**

```
The input file (NCPdata.xlsx) is exported from Questionnaire Star® and must contain the following 29 columns. Direct personal identifiers and potentially identifying fields (a6 IP Origin, x1 Name, x2 Phone Number, x3 ID Number, and the screenshot fields x8, x10a, x10b, and x17) are removed from the analytical dataset immediately after import (Module 3, Step 3a); the national identification number (x3) is used only to construct an anonymized deduplication key, and none of these fields are included in the summary tables or any exported output:
  a1     Index                          Sequential record identifier assigned by Questionnaire Star
  a2     Submission Time                Timestamp of form submission (YYYY-MM-DD HH:MM:SS)
  a3     Time Used                      Time spent completing the form (seconds)
  a4     Source                         Access channel (e.g., WeChat, direct link)
  a5     Source Details                 Detailed source information
  a6     IP Origin                      IP address of the submitter (removed during de-identification, Module 3 Step 3a)
  x1     Name                           Full name of the reporting individual
  x2     Phone Number                   Contact phone number
  x3     ID Number                      National identification number (unique key for deduplication)
  x4     Personnel Type                 Category: staff, student, third-party contractor, patient, family member
  x5     Department                     Hospital department of the reporting individual
  x6     Reporting Type                 Abnormality Reporting or other categories
  x7     Abnormality Description        Text description of the abnormality
  x8     Screenshot of Prompt Message   Uploaded image of the alert message (if applicable)
  x9     Health Code Status             Green, Yellow, or Red
  x10a   Health Code Screenshot         Uploaded health code image
  x10b   Health Code: Name              Auto-extracted name from health code OCR
  x10c   Health Code: Time              Auto-extracted timestamp from health code
  x10d   Health Code: Color             Auto-extracted color from health code
  x10e   Nucleic Acid Test Interval                   Nucleic acid test interval (hours since last test)
  x10f   Nucleic Acid Test Result                     Most recent nucleic acid test result
  x10g   Nucleic Acid Test Time                       Timestamp of most recent nucleic acid test
  x11    Community Requirements         Community-level reporting requirements
  x12    Residential Building Risk Level            Risk level of residence building (Low/Medium/High)
  x13    Close or Secondary Contact        Whether individual is a close or secondary contact
  x14    Estimated Completion Time      Estimated time to complete prevention-and-control requirements
  x15    Completion Time                Actual completion time of prevention-and-control requirements
  x16    Currently in Hospital          Whether the individual is physically present in the hospital
  x17    Risk-Clearance Certificate     Uploaded certificate of risk clearance (if applicable)
```

**4. Complete R Script**

The following script was executed daily during the deployment period (August 25–September 16, 2022). Comments (prefixed with #) document the purpose of each code block.

```
# ============================================================
# MODULE 1: Package Installation and Library Loading
# ============================================================

# Install required packages (run once per environment)
install.packages("Hmisc")
install.packages("lubridate")
install.packages("tidyverse")

# Load libraries
library(readxl)      # Reading Excel (.xlsx) files
library(Hmisc)       # Variable labeling utilities
library(lubridate)   # Date parsing and manipulation
library(tidyverse)   # Data wrangling (dplyr, tidyr, ggplot2, etc.)

# ============================================================
# MODULE 2: Data Import and Variable Labeling
# ============================================================

# Import data exported from Questionnaire Star
NCPdata <- read_excel("~/WorkSpace/testNCP/NCPdata.xlsx")

# Assign variable names to columns
names(NCPdata) <- c("a1", "a2", "a3", "a4", "a5", "a6", "x1", "x2",
                     "x3", "x4", "x5", "x6", "x7", "x8", "x9", "x10a",
                     "x10b", "x10c", "x10d", "x10e", "x10f", "x10g", "x11",
                     "x12", "x13", "x14", "x15", "x16", "x17")

# Define human-readable variable labels
label <- c("Index", "Submission Time", "Time Used", "Source", "Source Details",
           "IP Origin", "1. Name", "2. Please enter your phone number",
           "3. ID Number", "4. Personnel Type", "5. Department",
           "6. Reporting Type",
           "7. Abnormality Type and Description",
           "8. Screen Capture of Alert Message",
           "9. Health Code Status",
           "10. Health Code Screenshot: — Health Code",
           "10. Name", "10. Time", "10. Color",
           "10. Nucleic Acid Test Interval",
           "10. Nucleic Acid Test Result",
           "10. Nucleic Acid Test Time",
           "11. Community Reporting Requirements",
           "12. Residential Building Risk Level",
           "13. Are you a close or secondary contact? (Reconfirm)",
           "14. Estimated Completion Time of Prevention and Control Requirements",
           "15. Completion Time of Prevention and Control Requirements",
           "16. Current Hospital Attendance Status",
           "17. Risk-Clearance Certificate (if applicable)")

# Apply labels to all 29 variables
for (i in 1:29) {
  label(NCPdata[[i]]) <- label[[i]]
}

# ============================================================
# MODULE 3: Data Cleaning
# ============================================================
# --- Step 3a: Create an anonymized deduplication key and remove direct identifiers ---
# An anonymous key is derived from the national identification number (x3) BEFORE any
# identifying variables are removed; it is the only individual-level information retained
# in the analytical dataset.
NCPdata$dedup_key <- as.integer(factor(NCPdata$x3))

# Direct personal identifiers and potentially identifying fields (the submitter's IP address,
# name, phone number, national identification number, and uploaded screenshot fields) are removed from the analytical dataset as early as possible,
# so that no downstream step (filtering, deduplication, or summary generation) ever operates
# on identifiable information.
NCPdata <- select(NCPdata, -c(a6, x1, x2, x3, x8, x10a, x10b, x17))

# --- Step 3b: Validate and clean completion dates ---
# Missing or invalid completion dates are treated as missing (NA) and are NOT replaced with
# a placeholder; such records are retained in the analytical dataset.
NCPdata$x15 <- ymd(NCPdata$x15)

# --- Step 3c: Exclude records with implausible future completion dates ---
# A FIXED study-end cutoff date is used instead of Sys.Date(), so that re-running the script
# at any later time always yields identical results.
study_end_date <- as.Date("2022-09-16")  # end of the study observation window
NCPdata <- filter(NCPdata, is.na(x15) | x15 <= study_end_date)

# --- Step 3d: Filter for abnormality reports only (BEFORE deduplication) ---
# This ordering ensures that an abnormality report is not discarded by deduplication if an
# individual also submitted a routine (non-abnormality) report.
NCPdata <- filter(NCPdata, x6 == "Abnormality Reporting")

# --- Step 3e: Retain only the most recent record per individual ---
# Sort by Index in descending order (most recent submissions first).
NCPdata$a1 <- as.numeric(NCPdata$a1)
NCPdata <- NCPdata[order(-NCPdata$a1), ]

# Deduplicate by the anonymized key, keeping only the first (most recent) record.
NCPdata <- distinct(NCPdata, dedup_key, .keep_all = TRUE)

# Restore original ascending order and remove the temporary deduplication key.
NCPdata <- NCPdata[order(NCPdata$a1), ]
NCPdata <- select(NCPdata, -dedup_key)


# ============================================================
# MODULE 4: Automated Summary Table Generation
# ============================================================

# --- Summary Table 1: Health Code Status Distribution ---
# Counts of Green / Yellow / Red health codes among personnel at risk
mytable1 <- with(NCPdata, table(x9))
print("=== Health Code Status Distribution ===")
print(mytable1)

# --- Summary Table 2: Residential Building Risk Level Distribution ---
# Counts of Low / Medium / High risk-level residences
mytable2 <- with(NCPdata, table(x12))
print("=== Building Risk Level Distribution ===")
print(mytable2)

# --- Summary Table 3: Close / Secondary Contact Classification ---
# Counts of close contacts vs. secondary contacts
mytable3 <- with(NCPdata, table(x13))
print("=== Close / Secondary Contact Classification ===")
print(mytable3)

# ============================================================
# END OF SCRIPT
# ============================================================
# Total execution time: typically < 5 seconds for a daily batch of
# 100–500 records on a standard desktop computer
```

**5. Usage Notes**

• The input Excel file must be placed at the specified path (or the path updated in the read_excel() call).

• All direct personal identifiers and potentially identifying fields (the submitter's IP address, name, phone number, national identification number, and uploaded screenshots) are removed from the analytical dataset immediately after import (Module 3, Step 3a) and are never included in the summary tables or any output file. Processing occurs locally on a password-protected workstation within the hospital network; data are not transmitted outside the hospital network.

• The script is designed for batch processing and should be re-run each time new data are exported from Questionnaire Star® (typically daily during active outbreak periods).

• The output summary tables (mytable1–3) are printed to the R console; for production use, these may be exported to CSV or integrated into automated reporting dashboards.

**6. Data Availability**

The de-identified aggregated surveillance dataset supporting the findings of the associated manuscript is provided as Supplementary Material 2. Individual-level data containing personal identifiers cannot be shared due to privacy regulations.
